# Spontaneous Tumour Lysis Syndrome in Metastatic Endometrial Carcinoma

**DOI:** 10.7759/cureus.117379

**Published:** 2026-10-03

**Authors:** Katherine Coggins, Alice Irving

**Affiliations:** 1 Internal Medicine, Lincoln County Hospital, Lincoln, GBR

**Keywords:** cairo-bishop criteria, dedifferentiated endometrial sarcoma, endometrial sarcoma, gynecologic oncology, hyperuricaemia, oncological emergency, spontaneous tumour lysis syndrome, tumour lysis syndrome

## Abstract

Tumour lysis syndrome (TLS) is a potentially fatal oncological emergency characterised by rapid cellular breakdown resulting in hyperkalaemia, hyperphosphataemia, hyperuricaemia, and hypocalcaemia, with subsequent acute kidney injury, cardiac dysrhythmias, and multi-organ dysfunction. It most commonly occurs following initiation of anti-cancer therapy in highly proliferative haematological malignancies. Spontaneous tumour lysis syndrome (STLS), occurring in the absence of a therapeutic trigger, is considerably rarer, particularly in solid tumours.

We report a 55-year-old female presenting with progressive abdominal distension, pain, constipation, and oliguria progressing to anuria. Investigation demonstrated a 14 × 12 cm pelvic mass with extensive peritoneal and omental metastatic disease and large-volume ascites. During admission, prior to any anti-cancer therapy, she developed severe refractory hyperkalaemia, peaking at 8.1 mmol/L, alongside hyperphosphataemia >1.45 mmol/L (peaking at 2.44 mmol/L), hyperuricaemia of ≥476 µmol/L (peaking at 784 mmol/L), ionised hypocalcaemia ≤1.12 mmol/L (1.06 mmol/L), and rapidly progressive acute kidney injury, with creatinine rising from a baseline of 54 µmol/L to 310 µmol/L. These abnormalities fulfilled the Cairo-Bishop criteria for both laboratory TLS by achieving two or more deranged serum biochemical markers or changes >25% from baseline, as well as clinical TLS with the presentation of acute kidney injury and eventual death. Histopathological and genetic assessment ultimately identified a dedifferentiated endometrial carcinoma. Despite appropriate intravenous hydration, rasburicase, and repeated correction of electrolyte abnormalities, renal function and multi-organ dysfunction progressively deteriorated. The patient died nine days after admission, before anti-cancer therapy could be commenced. It was this absence of anti-cancer therapy and the trigger for the TLS that led us to classify this patient's presentation as STLS.

STLS is exceptionally uncommon in solid malignancies but may occur in aggressive tumours with a high disease burden and rapid cellular turnover. This case is particularly notable because biochemical evidence of TLS preceded biopsy and any oncological treatment, supporting a genuinely spontaneous presentation. Furthermore, conventional risk stratification classified this patient as low risk, demonstrating the limitations of existing approaches when applied to rare, aggressive solid malignancies.

STLS should be considered in patients with newly diagnosed or suspected solid malignancy who develop otherwise unexplained hyperkalaemia, hyperphosphataemia, hyperuricaemia, hypocalcaemia, or acute kidney injury, even before anti-cancer treatment. Early recognition and application of TLS diagnostic criteria are essential to facilitate prompt hydration, urate-lowering therapy, and correction of life-threatening metabolic abnormalities; however, it is key to note that, despite this, this condition remains to possess a poor prognosis. Greater awareness of STLS in aggressive solid tumours may enable earlier intervention and potentially improve outcomes for this fulminant condition.

## Introduction

Tumour lysis syndrome (TLS) is a significant oncological emergency. Physiologically, it involves the sudden and extensive catabolism of cancer cells, usually following oncological treatment in bulky haematological malignancies [1]. However, spontaneous TLS (STLS), where TLS occurs without any precipitating factor, is rare. The characteristic biochemical derangement (namely, hyperphosphataemia, hyperuricaemia, hyperkalaemia, and hypocalcaemia) forms the basis of the pathophysiological consequences: acute kidney injury (AKI), cardiac dysfunction, neuromuscular irritability, and other systemic inflammatory reactions [2]. Documentation of STLS cases aims to highlight this presentation in at-risk patients, both with or without precipitating factors, to achieve early initiation of treatment and improve mortality rates. Raising awareness also alerts clinicians to STLS as a potential first presentation of malignancy in the undiagnosed cancer patient. In this article, we present a rare case of a 55-year-old female with newly diagnosed stage IV metastatic complex endometrial carcinoma, primarily presenting as an ovarian mass, who developed STLS prior to any oncological treatment.

## Case presentation

This case focuses on a 55-year-old non-parous female who presented to the emergency department following a three-week history of constipation (five days, absolute), abdominal distension, worsening abdominal pain, oliguria (two days), and anuria (one day). She had one episode of vomiting three days prior to admission. On examination, she had a distended, tense abdomen and moderate ascites with a positive fluid shift.

Her past medical history included multiple sclerosis, endometriosis, mitral valve regurgitation, and cervicothoracic scoliosis. Gynaecologically, she suffered from endometriosis, adhesions, and bilateral chocolate cysts, investigated with a diagnostic laparotomy 10 years previously. She had no smoking history but had a previous alcohol history of 56 units per week; she had been sober for many years. She did not take any regular medications. She reported a maternal history of ovarian cancer but no other familial malignancies.

The patient's admission bloods revealed severe hyperkalaemia, peaking at 8.1 mmol/L during admission prior to any imaging and procedural interventions. This developed into refractory hyperkalaemia, with multiple treatment bundles of IV insulin, dextrose, salbutamol, calcium gluconate, and sodium zirconate providing only transient reprieve. She was also found to have a serum CA-125 level of 926. During admission, her blood tests revealed various progressive electrolyte derangements, including hyperkalaemia, hyperphosphataemia, hyperuricaemia, and hypocalcaemia, as seen in Figure 1. Throughout the admission, she developed a worsening AKI, with her glomerular filtration rate (GFR) falling from 86 ml/min/1.73 m^2^ to 14 ml/min/1.73 m^2^ and creatinine rising >6x her usual baseline of 54 umol/L.

**Figure 1:**
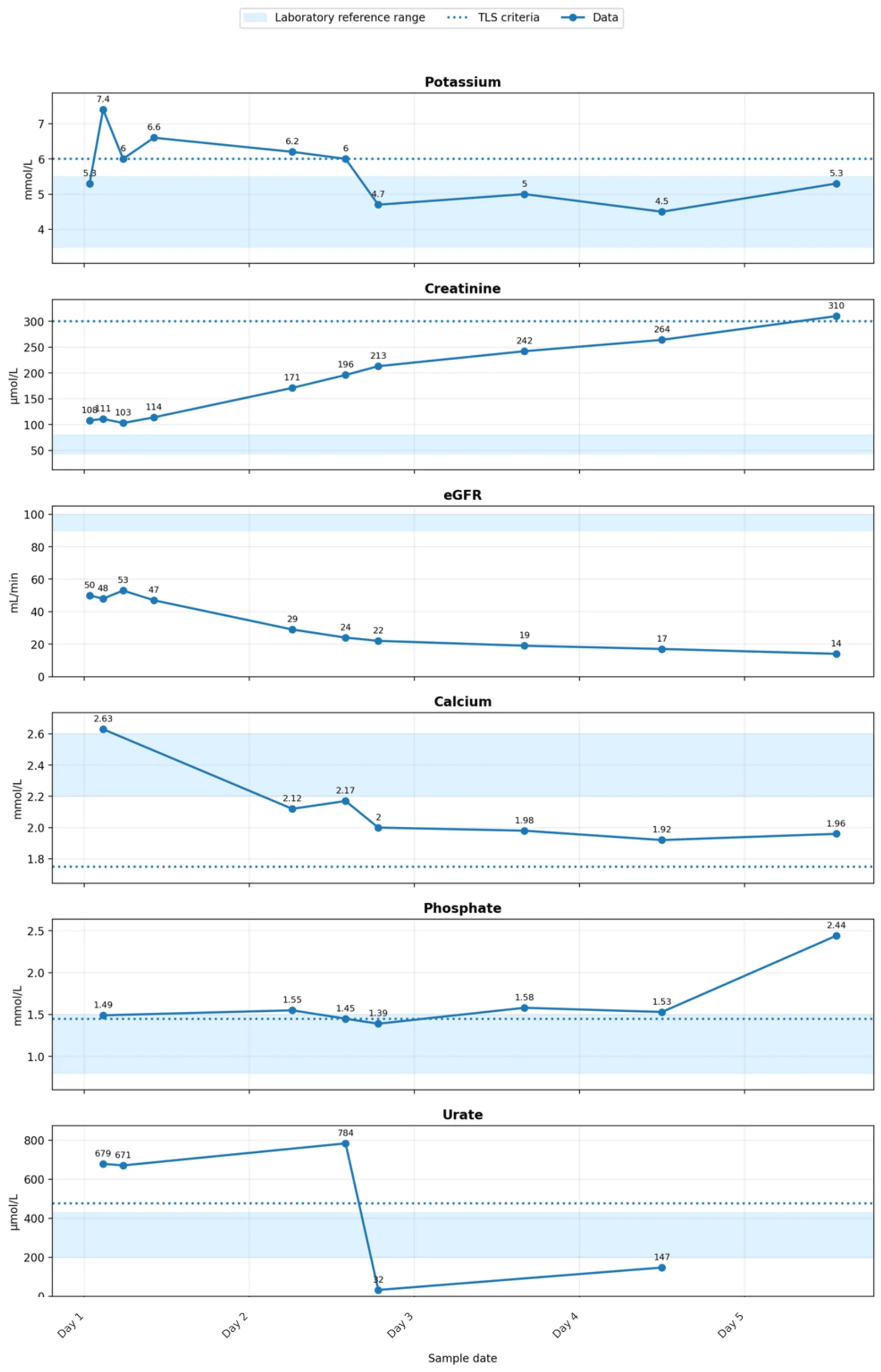
A compound line graph showing the evolution of biochemical markers, including potassium, creatinine, eGFR, phosphate, urate, and calcium of the patient throughout admission, as well as the laboratory reference ranges and associated TLS criteria values. TLS: tumour lysis syndrome; eGFR: estimated glomerular filtration rate.

In this patient’s case, these electrolyte presentations fulfilled the diagnostic criteria for TLS in keeping with the Cairo-Bishop criteria of two or more specific electrolyte abnormalities [1]. Across a three-day period, the patient’s biochemical results showed a urate level >476 µmol/L, a potassium level >6 mmol/L, and a phosphate level >1.45 mmol/L, as seen in Figure 1. The patient did not exhibit a significant hypocalcaemia to reach the <1.75 mmol/L target or more than 25% change from baseline for TLS; however, on a venous blood gas test on day 5, we did record an ionised calcium level of 1.06 mmol/L, which did fulfil the target of <1.12 mmol/L for ionised calcium levels [1]. Despite serum calcium not fulfilling the TLS criteria, it is clear in Figure 1 that her serum calcium levels were continuously trending down. Regarding her diagnosis, she also achieved the status of clinical TLS with the development of her AKI during her admission, where her creatinine peaked at 310 µmol/L, and her estimated glomerular filtration rate (eGFR) remained consistently low. Clinically, she did not develop any cardiac arrhythmia or have any seizure activity documented; however, ECG changes consistent with hyperkalaemia were present.

Following this, she underwent various imaging modalities. A contrast-enhanced computed tomography (CT) of her abdomen and pelvis revealed a large heterogeneous solid-mass lesion seen in the central pelvis, likely adnexal in origin, measuring approximately 14 x 12 cm, as seen in Figure 2. There was extrinsic compression of the rectosigmoid by this mass lesion and extensive peritoneal and omental deposits with large-volume ascites. There was also an enlarged aortocaval node seen measuring 2.7 cm in short-axis diameter.

**Figure 2:**
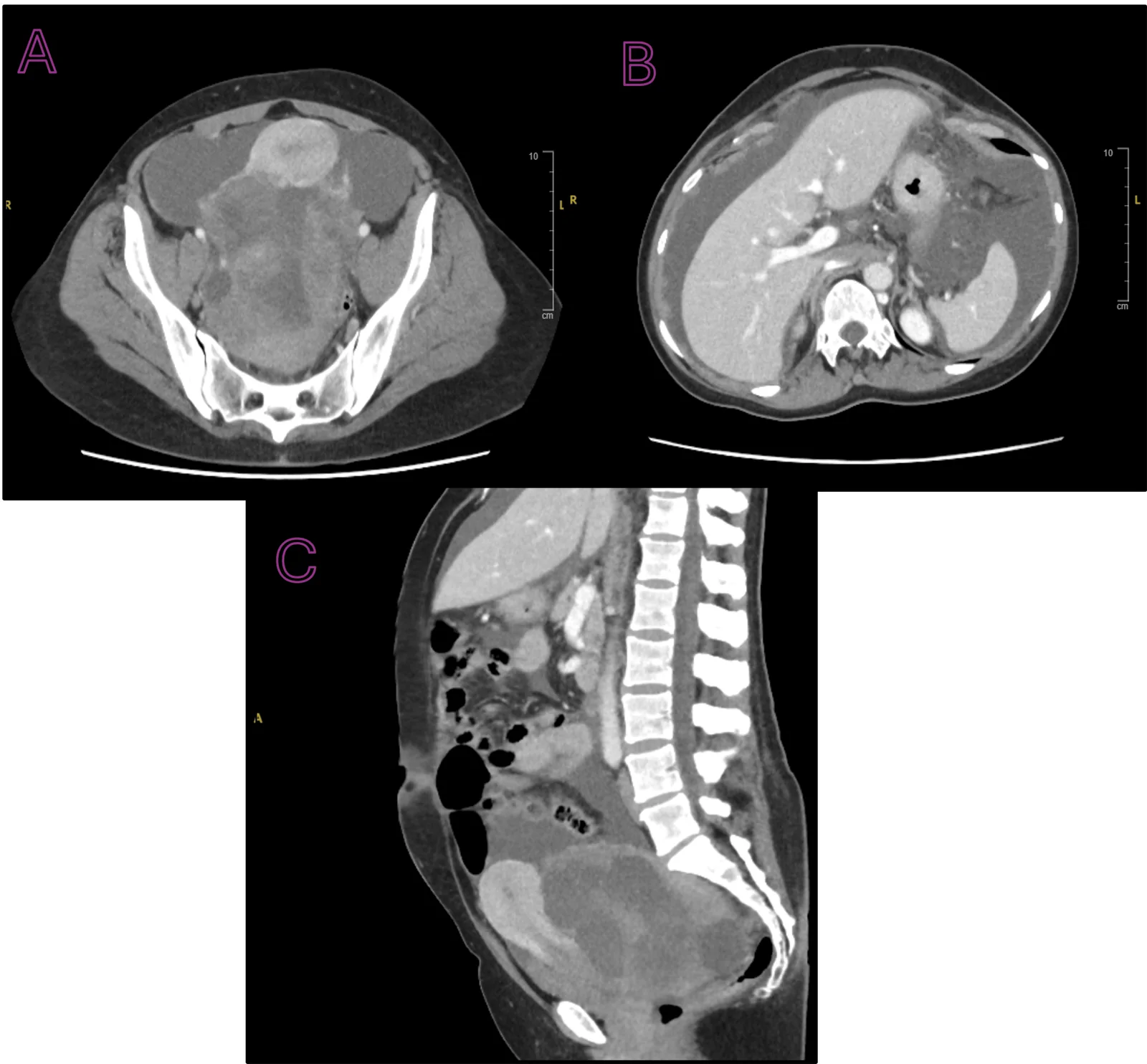
Contrast-enhanced CT imaging demonstrating advanced pelvic malignancy. A: Large heterogeneous adnexal mass. B: Large-volume ascites. C: Coronal view demonstrating tumour size and mass effect.

Due to the extent of the ascites and associated abdominal compression, the patient had an ultrasound-guided ascitic drain inserted, draining more than 10 litres of ascitic fluid, and biopsies of the omental deposits. Following this, she had a staging contrast CT of her chest, revealing a few prominent pericardiac/left cardiophrenic lymph nodes. During this period, the patient exhibited adequate renal function to warrant a contrast-enhanced CT scan; however, due to the nature of TLS and pathophysiological tendency towards AKI, the contrast status of the imaging needs to be evaluated on an individual basis.

Histological investigations identified the biopsy samples initially as undifferentiated small blue round cell neoplasm, most consistent with small round cell sarcoma. Further fluorescence in situ hybridisation studies excluded Ewing sarcoma, endometrial stromal sarcoma, and N-MYC-amplified neuroblastoma. For diagnostic clarification, the sample underwent pan-cancer gene panel RNA analysis showing biallelic inactivation of PTEN and ARID1A with KRAS mutations and possible biallelic loss of ARID1B. This resembled epithelial origin and is characteristic of high-grade Mullerian carcinoma consistent with endometrioid origin. However, with no TP53 mutations identified and combined with the molecular and morphological findings, the consensus genetic diagnosis was a dedifferentiated/undifferentiated endometrial carcinoma.

The patient initially received aggressive intravenous fluid resuscitation; however, as a result of her progressive intrinsic renal damage, whereby the patient entered from an oliguric into an anuric state, the use of intravenous fluids to help maintain renal perfusion and excretion of excessive metabolites became more harmful in terms of risking circulatory overload and pulmonary oedema. These were stopped once their administration was felt to be of no physiological benefit to the patient. During admission, a further non-contrast CT kidney-ureter-bladder scan excluded any obstructive uropathy, supporting intrinsic renal injury secondary to TLS. Other treatments she received included electrolyte correction and rasburicase once TLS had been identified. Despite this, her renal function progressively declined (eGFR reduced to 14 ml/min/1.73 m^2^) with further multi-organ dysfunction. The patient died nine days after her admission, before any anti-cancer therapy could be started.

Pathophysiology

The biochemical metabolites released during the lysis of cancer cells provide the pathophysiological foundation for the presentation of TLS. It is this excessive release of electrolytes that overwhelms our normal homeostatic mechanisms. During direct cell lysis, there is a profound release of potassium and phosphate, whilst simultaneously the metabolic breakdown of endogenous nucleic acids and purines from other components of an apoptotic cell causes a build-up of uric acid. Each of these electrolyte derangements has its own physiological consequences.

Hyperkalaemia induces gastrointestinal issues, namely, nausea and vomiting, muscle weakness, as well as fatal dysrhythmias. Hyperphosphataemia also increases the risk of dysrhythmias as well as nephrocalcinosis, contributing to the development of AKI. It elicits these effects due to the sequestration of calcium into calcium-phosphate complexes, which can deposit in soft tissues, including cardiac myocytes and nephrons [2]. Hypocalcaemia caused by this complex formation can further lead to mental health disturbances, muscle tetany, dysrhythmias, and seizures [3].

Hyperuricaemia is usually attributed to inadequate renal excretion; however, in TLS, it is the sheer excess of production of uric acid that renders renal excretion unable to maintain homeostasis [4]. Exacerbation of this excretory insufficiency occurs due to formation of intrarenal urate crystallisation, causing both obstruction of renal blood flow as well as intrarenal inflammation [3]. The urate crystals can then precipitate in urine and contribute to urine retention and obstructive uropathic AKI. With further progression of this renal damage and limited means of metabolic excretion of not only uric acid but also potassium and phosphate, the metabolic load and physiological damage can exponentially increase.

In addition to these metabolic disturbances, the extensive cell lysis promotes release of pro-inflammatory cytokines that contribute to systemic inflammatory processes [5]. The dysregulated cytokine activity can affect endothelial function and vascular tone, which can contribute to haemodynamic instability and multi-organ dysfunction [5]. This can be considered a secondary consequence of the primary lytic process rather than a defining feature of TLS.

The trigger for this mass cellular lysis is usually anti-cancer therapy (chemotherapy, radiotherapy, or glucocorticoid therapy); however, in rare reports, cancer cells can spontaneously lyse with no obvious trigger [6]. In these instances, the term "spontaneous tumour lysis syndrome" (STLS) can be applied. There are cases in which cell lysis has occurred shortly after biopsies were taken, resulting in procedure-induced STLS. However, in the case of this patient, she has started exhibiting electrolyte derangement patterns consistent with STLS prior to her omental biopsy [7]. Nevertheless, the potential for this structural disruption to trigger TLS needs to be communicated to patients, and clinicians need to be made more aware of this risk across low- and high-risk patient groups.

The underlying mechanism of STLS is not fully understood, but it is thought to involve a rapid and unstable tumour cell turnover rate, combined with insufficient substrate supply to sustain this level of cellular proliferation. This imbalance may then result in widespread apoptotic cell death and subsequent intracellular electrolyte release [8]. Although not well understood, there are groups of malignancies that seem to present with TLS more commonly, with haematological malignancies being the main culprit [6-8]. However, they are not the only cause; any cancerous tumour has the potential to lyse, especially after starting anti-cancer treatment, including gynaecological malignancies [9].

In this case report, it was an undifferentiated/dedifferentiated endometrial carcinoma that was the source of the STLS. These are exceptionally rare, highly aggressive uterine cancers characterised by rapid progression and poor prognosis. Owing to this rarity, published outcome data are limited; however, advanced-stage disease is understandably associated with profoundly reduced survival, with reported five-year overall survival rates of approximately 12-25% for stage IV disease [10,11]. These tumours frequently present at an advanced stage due to their aggressive nature and significant metastatic capabilities.

Diagnostic criteria

TLS has two major diagnostic subgroups, laboratory TLS and clinical TLS, commonly classified by the Cairo and Bishop criteria [1]. When diagnosing laboratory TLS, two or more deranged serum biochemical parameters or a 25% change from baseline over a three-day period, usually three days before or seven days after starting oncological therapy, must be present [1]. These laboratory abnormalities include hyperphosphataemia ≥1.45 mmol/L, hyperuricaemia >476 µmol/L, hyperkalaemia ≥6 mmol/L, and hypocalcaemia ≤1.75 mmol/L [1].

Clinical TLS diagnosis requires the presence of laboratory TLS, plus one of the following features: cardiac dysrhythmia, seizures, AKI (increased creatinine level >1.5x baseline or 0.3 mg/dl), or sudden death [1]. Further review by Howard et al. stipulated that any symptomatic hypocalcaemia in the context of TLS should also constitute a diagnosis [3].

In the case of this patient, she met the criteria for both laboratory and clinical TLS owing to her significant biochemical derangement and AKI (Table 1). Despite the lack of seizure activity and on account of the TLS resulting in the patient's death, her case can be classified as a grade 5 STLS in keeping with the Cairo-Bishop criteria (Table 2) [1].

**Table 1:** Application of the Cairo-Bishop diagnostic criteria to the patient. Cairo-Bishop diagnostic criteria [1]. eGFR: estimated glomerular filtration rate.

Diagnostic criterion	Patient finding	Criterion met
Potassium ≥6.0 mmol/L	8.1 mmol/L	✓
Phosphate ≥1.45 mmol/L	1.5 mmol/L	✓
Serum calcium ≤1.75 mmol/L	1.91 mmol/L	x
Ionised calcium (Ca^2+^) <1.12 mmol/L	1.06 mmol/L	✓
Urate ≥476 µmol/L	784 umol/L	✓
Acute kidney injury	Creatinine rise/eGFR = 14 mL/min/1.73 m²	✓
Cardiac arrhythmia	No documented arrhythmia (ECG changes present)	✗
Overall diagnosis	Clinical spontaneous tumour lysis syndrome	

**Table 2:** TLS grades from the Cairo-Bishop criteria. TLS: tumour lysis syndrome; ULN: upper limit of normal; Cr: creatinine. Adapted from Cairo and Bishop [1]. Used with permission from John Wiley & Sons. Permission conveyed through Copyright Clearance Center (Danvers, MA).

Grade	Clinical features	Definition
Grade 0	Laboratory TLS only (no clinical criteria).	No clinical manifestations.
Grade 1	• Cr 1.5× ULN • Cardiac arrhythmia not requiring urgent intervention • No seizures	Laboratory TLS with mild clinical manifestations not requiring urgent intervention.
Grade 2	• Cr >1.5–3.0× ULN • Non-urgent medical intervention for arrhythmia • Brief generalized seizure	Moderate clinical manifestations requiring non-urgent medical intervention.
Grade 3	• Cr >3.0–6.0× ULN • Symptomatic/severe arrhythmia • Prolonged seizure	Severe clinical manifestations requiring urgent intervention.
Grade 4	• Cr >6.0× ULN • Life-threatening arrhythmia • Life-threatening seizure	Life-threatening clinical manifestations requiring immediate intervention.
Grade 5	• Death	Death. TLS is the direct cause of death.

Risk stratification

The risk of developing TLS relies on tumour-dependent and tumour-independent factors. Tumours that rapidly proliferate and have a high tumour burden and chemosensitivity are well-documented factors increasing the likelihood of TLS [12]. In 2010, an expert panel proposed a risk stratification model that accounts for tumour type, white cell count, histological diagnosis, disease burden, age, anti-cancer therapy, and renal function [13,14]. They classified virtually all solid tumours as low risk of TLS occurrence (<1%) while chemo-sensitive or bulky solid tumours as intermediate risk [14]. High risk was defined as more than 5%, which involves some haematological malignancies, including Burkitt lymphoma [14]. As our case confirms, even conventionally identified as low-risk malignancies, such as a solid endometrial carcinoma, can develop into TLS and require intensive therapy.

Patient-specific risk factors included pre-existing renal disease, hypovolaemia or hypotension, pre-treatment hyperphosphataemia or hyperuricaemia, and acidic urine [15]. If patients are high risk, then increased monitoring, hydration as appropriate, and prophylactic rasburicase are used to mitigate risk; however, this is not necessary for low- or intermediate-risk patients. Allopurinol should be offered to low-risk patients at the initiation of treatment; however, this only manages the hyperuricaemia effects of TLS [16]. Hyperkalaemia, hypocalcaemia, and hyperphosphataemia have to be managed clinically via alternative methods.

The patient in this case was at low risk according to the criteria. Unfortunately, the patient's malignancy was an incidental finding and her progression to STLS was rapid. This rate of deterioration highlights the devastating nature of oncological emergencies within this vulnerable patient group.

Treatment

Initially, the aim of treatment is to restore euvolaemic status in these patients and mitigate renal hypoperfusion. The recommended target of 3 L/24 hours is advised, but aggressive fluid hydration can be assessed on individual requirement and response levels [17]. This should be individualised based on volume status, urine output, renal function, and cardiac function. Due to the established pathophysiological processes affecting renal function, it is key to establish urine output so as not to precipitate volume overload and pulmonary oedema in patients with no physiological outlet for aggressive hydration. In these cases, renal replacement therapy should be discussed. In the case of our patient, such renal replacement therapy was not deemed appropriate due to the extensive metastatic late presentation of her malignancy.

The next focus is AKI and hyperuricaemia; rehydration provides a dilutional therapy against the electrolyte imbalances seen in AKI (hyperuricaemia, hyperphosphataemia, and hyperkalaemia). To treat the established hyperuricaemia, intravenous rasburicase is recommended. This acts as a recombinant urate oxidase to sequester uric acid into allantoin [18]. Phosphate-binding agents can also be introduced based on the severity of hyperphosphataemia to reduce the risk of further irreversible intrinsic complex-driven kidney damage. Although reactive therapy is still applicable, early detection of risk factors that may predispose patients to TLS can facilitate early use of allopurinol as a preventative strategy for hyperuricemia. This acts by inhibiting xanthine oxidase to reduce formation of new uric acid; however, it does not tackle pre-existing uric acid levels, and so for established hyperuricaemia, rasburicase is preferred over the preventative allopurinol [3].

Treatment of high phosphate helps to initiate homeostasis of calcium levels; however, in severe cases, where patients become symptomatic secondary to hypocalcaemia (i.e., seizures, tetany, or cardiac arrhythmias), infusion of calcium gluconate can be started under close monitoring to prevent aggressive calcium-phosphate precipitation in the kidneys if calcium is replaced too rapidly.

Management of TLS fundamentally revolves around aggressive hydration, hypouric drug therapy, and re-establishing electrolyte homeostasis [18]. As per guidelines, hyperkalaemia is treated with IV insulin, dextrose, salbutamol, calcium gluconate, and potassium-sequestering agents. In severe cases, haemofiltration is recommended [18]. During this treatment period, serial ECGs may be required depending on the clinical presentation and haemodynamic stability of the patient.

## Discussion

TLS is uncommon in solid tumour malignancies, and STLS is particularly rare. It is traditionally associated with highly proliferative haematological malignancies, particularly following the initiation of anti-cancer therapy [6,12]. However, this case demonstrates a presentation of STLS in aggressive metastatic solid tumours, specifically undifferentiated/dedifferentiated endometrial carcinoma, before any anti-cancer treatment has commenced. The precise mechanism underlying STLS remains unclear. Without any external trigger, those malignancies with especially rapid cellular turnover may exceed the available metabolic supply of the area, potentially resulting in cellular ischaemia and subsequent release of intracellular electrolytes [19]. In the case of this patient, her extensively metastatic status represents a high tumour burden with likely rapid cell turnover, which provides a plausible environment in which STLS can originate.

The rarity of STLS in solid malignancies creates a risk of delayed recognition, particularly when a patient has not yet received chemotherapy. In this case, progressive metabolic derangement and AKI occurred in the absence of a conventional therapeutic trigger. This highlights the importance of recognising the biochemical pattern of TLS irrespective of tumour type or treatment status. Unexplained hyperkalaemia, hyperphosphataemia, hypocalcaemia, hyperuricaemia, and AKI in a patient with a bulky or rapidly proliferating malignancy should therefore prompt consideration of TLS and early application of the Cairo-Bishop diagnostic criteria [1].

An additional consideration is the potential relationship between invasive tumour procedures and TLS. Previous reports have described STLS occurring following biopsy, while metastatic tumours may demonstrate increased cellular instability [20]. Importantly, the biochemical abnormalities in this patient were present prior to biopsy, supporting a genuinely spontaneous presentation rather than biopsy-induced TLS. Nevertheless, these reports raise the possibility that invasive procedures could further precipitate tumour lysis in susceptible patients. Greater awareness of this potential complication may therefore be warranted when investigating patients with bulky or highly aggressive metastatic malignancies, including endometrial carcinoma.

Prevention and early intervention remain central to reducing morbidity and mortality associated with TLS. Established preventative strategies include appropriate risk stratification, hydration, biochemical monitoring, and urate-lowering therapy in patients considered at increased risk [14]. However, conventional risk stratification may fail to identify some patients with aggressive solid malignancies, as demonstrated by this case. Whether selected patients with high-burden metastatic solid tumours would benefit from additional monitoring or prophylactic treatment surrounding invasive procedures remains unclear. Further research is required to establish whether tumour burden, metastatic disease, and tumour biology should be given greater consideration when assessing TLS risk in solid malignancies. Regardless of this, extensive awareness of the severity of this condition and its capability to originate from many solid tumours is vital to our ability to resolve it.

## Conclusions

STLS is a rare but potentially catastrophic oncological emergency that should not be considered exclusive to haematological malignancies or anti-cancer therapy. This case demonstrates that STLS can occur spontaneously in an aggressive metastatic gynaecological malignancy. It also highlights that despite recognition of STLS and appropriate interventions, the patient continued to have a rapidly progressing severe metabolic disturbance, AKI, and multi-organ dysfunction. This meant that, despite therapy, the patient died nine days after admission.

Clinicians should maintain a high index of suspicion for TLS when patients with bulky or rapidly proliferating malignancies develop otherwise unexplained hyperkalaemia, hyperphosphataemia, hyperuricaemia, hypocalcaemia, or AKI, regardless of tumour type or treatment status. Early recognition, prompt application of diagnostic criteria, and timely initiation of appropriate management are essential to ultimately help improve patient outcomes; however, as demonstrated by this case, STLS in an aggressive metastatic tumour may follow a fulminant course with a poor prognostic outcome despite appropriate management. This case also highlights a limitation of current risk stratification models when applied to rare aggressive solid malignancies. Greater clinical awareness of STLS may help facilitate earlier recognition and intervention in patients who would otherwise be considered at low risk.
